# Trajectories of interleukin 10 and heart fatty acid-binding protein levels in traumatic brain injury patients with or without extracranial injuries

**DOI:** 10.3389/fneur.2023.1133764

**Published:** 2023-04-04

**Authors:** Toni J. U. Niiranen, Anne-Cécile Chiollaz, Riikka S. K. Takala, Miko Voutilainen, Olli Tenovuo, Virginia F. J. Newcombe, Henna-Riikka Maanpää, Jussi Tallus, Mehrbod Mohammadian, Iftakher Hossain, Mark van Gils, David K. Menon, Peter J. Hutchinson, Jean-Charles Sanchez, Jussi P. Posti

**Affiliations:** ^1^Department of Clinical Neurosciences, University of Turku, Turku, Finland; ^2^Department of Medicine, Faculty of Medicine, University of Geneva, Geneva, Switzerland; ^3^Perioperative Services, Intensive Care Medicine, and Pain Management, Turku University Hospital and University of Turku, Turku, Finland; ^4^Anaesthesiology, Intensive Care, Emergency Care and Pain Medicine, University of Turku, Turku, Finland; ^5^Department of Microbiology, Faculty of Agriculture and Forestry, University of Helsinki, Helsinki, Finland; ^6^Turku Brain Injury Center, Turku University Hospital, Turku, Finland; ^7^Division of Anaesthesia, Addenbrooke’s Hospital, University of Cambridge, Cambridge, United Kingdom; ^8^Department of Radiology, Turku University Hospital, Turku, Finland; ^9^Neurocenter, Department of Neurosurgery, Turku University Hospital, Turku, Finland; ^10^Faculty of Medicine and Health Technology, Tampere University, Tampere, Finland; ^11^Department of Clinical Neurosciences, Neurosurgery Unit, Addenbrooke’s Hospital, University of Cambridge, Cambridge, United Kingdom

**Keywords:** traumatic brain injury, interleukin-10, H-FABP, extracranial injury, C-reactive protein

## Abstract

**Background:**

Interleukin 10 (IL-10) and heart fatty acid-binding protein (H-FABP) have gained interest as diagnostic biomarkers of traumatic brain injury (TBI), but factors affecting their blood levels in patients with moderate-to-severe TBI are largely unknown.

**Objective:**

To investigate the trajectories of IL-10 and H-FABP between TBI patients with and without extracranial injuries (ECI); to investigate if there is a correlation between the levels of IL-10 and H-FABP with the levels of inflammation/infection markers C-reactive protein (CRP) and leukocytes; and to investigate if there is a correlation between the admission level of H-FABP with admission levels of cardiac injury markers, troponin (TnT), creatine kinase (CK), and creatine kinase MB isoenzyme mass (CK-MBm).

**Materials and methods:**

The admission levels of IL-10, H-FABP, CRP, and leukocytes were measured within 24 h post-TBI and on days 1, 2, 3, and 7 after TBI. The admission levels of TnT, CK, and CK-MBm were measured within 24 h post-TBI.

**Results:**

There was a significant difference in the concentration of H-FABP between TBI patients with and without ECI on day 0 (48.2 ± 20.5 and 12.4 ± 14.7 ng/ml, *p* = 0.02, respectively). There was no significant difference in the levels of IL-10 between these groups at any timepoints. There was a statistically significant positive correlation between IL-10 and CRP on days 2 (R = 0.43, *p* < 0.01) and 7 (R = 0.46, *p* = 0.03) after injury, and a negative correlation between H-FABP and CRP on day 0 (R = -0.45, *p* = 0.01). The levels of IL-10 or H-FABP did not correlate with leukocyte counts at any timepoint. The admission levels of H-FABP correlated with CK (R = 0.70, *p* < 0.001) and CK-MBm (R = 0.61, *p* < 0.001), but not with TnT.

**Conclusion:**

Inflammatory reactions during the early days after a TBI do not significantly confound the use of IL-10 and H-FABP as TBI biomarkers. Extracranial injuries and cardiac sources may influence the levels of H-FABP in patients with moderate-to-severe TBI.

## Introduction

Traumatic brain injury (TBI) is a heterogeneous disease with complex pathophysiology. The acute diagnosis of TBI is based on symptoms and clinical signs as well as imaging findings on head computed tomography (CT). Based on these, TBIs have been traditionally classified as mild (mTBI), moderate (moTBI), and severe (sTBI) TBIs. After the initial insult neuroinflammatory processes may occur which may worsen the secondary injury (1).

Neuroinflammation is associated with the sequela of TBI, and it is associated with chronic traumatic encephalopathy and accelerated neurodegeneration (2). Mediators of neuroinflammation may be divided into proinflammatory (for example IL-Iβ, IL6, and IL17) and anti-inflammatory (for example IL-4, IL-10, IL-13) cytokines (1). IL-10 as an anti-inflammatory cytokine promotes the resolution of inflammatory cascades and some preclinical studies suggest that systemic IL-10 treatment may be beneficial for improving outcome after TBI (3).

IL-10 is expressed mainly in macrophages and B cells (4). IL-10 blood levels increase after a TBI of all severities (5). In patients with mTBI, IL-10 is a potential biomarker differentiating CT-positive patients from CT-negative and an early outcome predictor (6). In patients without ECI, IL-10 levels peak in the first 24 h after trauma decreasing in the following 4–5 days (7).

H-FABP is a cytoplasmic protein expressed in cardiac myocytes, making it a sensitive biomarker for myocardial infarction (8). H-FABP is also expressed in neurons and its blood levels increase after TBI (9). It is also a potential biomarker in the diagnostics of mTBI and in differentiating between CT-positive and CT-negative patients with mTBI (10). H-FABP levels in the blood peak 2–3 h after an ischemic stroke and remain steady for many days, making H-FABP a potential biomarker in brain insults (11).

C-reactive protein (CRP) is an acute phase protein and sensitive biomarker of systemic inflammation, which often increases in proportion to injury severity (12). Leucocytes are also involved in the acute phase of inflammation/infection (13). Therefore, it is important to investigate the correlation between CRP/leucocytes and IL-10/H-FABP to see if the inflammation associated with TBI disrupts the use of these biomarkers in the diagnostics of TBI.

Creatine kinase (CK) and Troponin T (TnT) are proteins that are primarily found in the heart cardiomyocytes and skeletal muscle (14). H-FABP, respectively, is found in both brain and heart making it important to evaluate the possible cardiac involvement in H-FABP levels.

Previous research on biomarkers of TBI has mainly focused on brain-enriched astroglial and neuronal/axonal proteins. So far, the combination of ubiquitin C-terminal hydrolase-L1 (UCH-L1) and glial fibrillary acidic protein (GFAP) has been approved by the US Food and Drug Administration (FDA) for clinical use in mTBI patients (15). IL-10 and H-FABP may be promising in the acute diagnostics and outcome prediction of TBI (5). This study continues our previous line of research on these biomarkers (5, 9, 16). Before applying biomarkers for clinical use in TBI, factors that may influence their levels must be understood and controlled for in various clinical settings.

The aim of this study was to investigate the trajectories of IL-10 and H-FABP in peripheral blood during the first week after TBI in mo/sTBI. The first aim of the study was to examine if the levels of IL-10 and H-FABP differ between patients with and without ECI. Moreover, our secondary aim was to determine whether their levels correlate with established clinical biomarkers of inflammation and cardiac injury. Therefore, we studied if the CRP and leukocyte levels correlate with IL-10 or H-FABP and if TnT and CK/CK-MBm levels correlate with the levels of H-FABP at admission.

## Materials and methods

### Study population

Patients with TBI were recruited during the EU-funded TBIcare (Evidence-based Diagnostic and Treatment Planning Solution for Traumatic Brain Injuries, EU FP7 Grant Agreement 270,259) prospective study between November 2011 and October 2013 at the Turku University Hospital, Finland.

Inclusion criteria for the TBI group were: moTBI or sTBI [worst Glasgow Coma Score (GCS) before possible intubation <13], age ≥ 18 years, and a clinical diagnosis of TBI with indications for acute head CT according to National Institute for Health and Care Excellence criteria (17). Exclusion criteria were head injury without an indication for CT, a prior neurological disease that causes an inability for independent living, penetrating or blast-induced injuries, chronic subdural hematoma, no consent obtained, or inability to speak Finnish. All patients or their proxies were given oral and written information about the study and written consent was obtained. Southwest Finland Hospital District Research Ethics Committee (decision 68/180/2011) approved the study.

The initial GCS was determined by paramedics at the accident scene, during hospital transport, or at the emergency department by an emergency physician. The lowest GCS before possible intubation was used in the demographics. For the analyses, patients were classified according to GCS. A TBI patient with or without a skull fracture was categorized as a TBI patient without ECI. A TBI patient with any other injuries than a skull fracture was categorized as a TBI patient with ECI. The total injury burden was assessed using the Injury Severity Score (ISS) (18). Helsinki CT Score (HCTS) was developed for outcome prediction in patients with TBI (19) and it was used in a multivariable logistic regression analysis.

### Biomarker analysis

First serum samples for IL-10 and H-FABP were obtained within 24 h from admission. After obtaining blood samples, they were centrifuged and then stored at −70°C. Blood samples were collected also on days 1, 2, 3, and 7 post-TBI. IL-10 and H-FABP were measured using V-plex Human IL-10 kit (K151QUD), and R-plex Human FABP3/H-FABP Antibody Set (F214T) on Meso Scale Platform (Meso Scale Diagnostics, Rockville, MD, USA). For the validated V-plex IL-10 assay, LLoD (Lower limit of detection) was 0.04 pg./ml and LLoQ (Lower limit of quantification) was 0.298 pg./ml. For the research R-plex H-FABP assay, LLoD was 0.90 ng/ml with a calibration range of 0.024–100 ng/ml and the calculated LLoQ was 0.390 ng/ml. CRP was collected in Li-heparin tubes and analyzed immunohistochemically. Leukocytes were collected in EDTA-tubes and analyzed using an automatized cell counter. TnT and CK-MBm were collected in Li-heparin tubes and analyzed with electrochemiluminescence (ECLIA). CK was collected in Li-heparin tubes and analyzed photometrically. All the kits were used as stated in the manufacturers’ guidance.

### Statistical analysis

**Figure 1:**
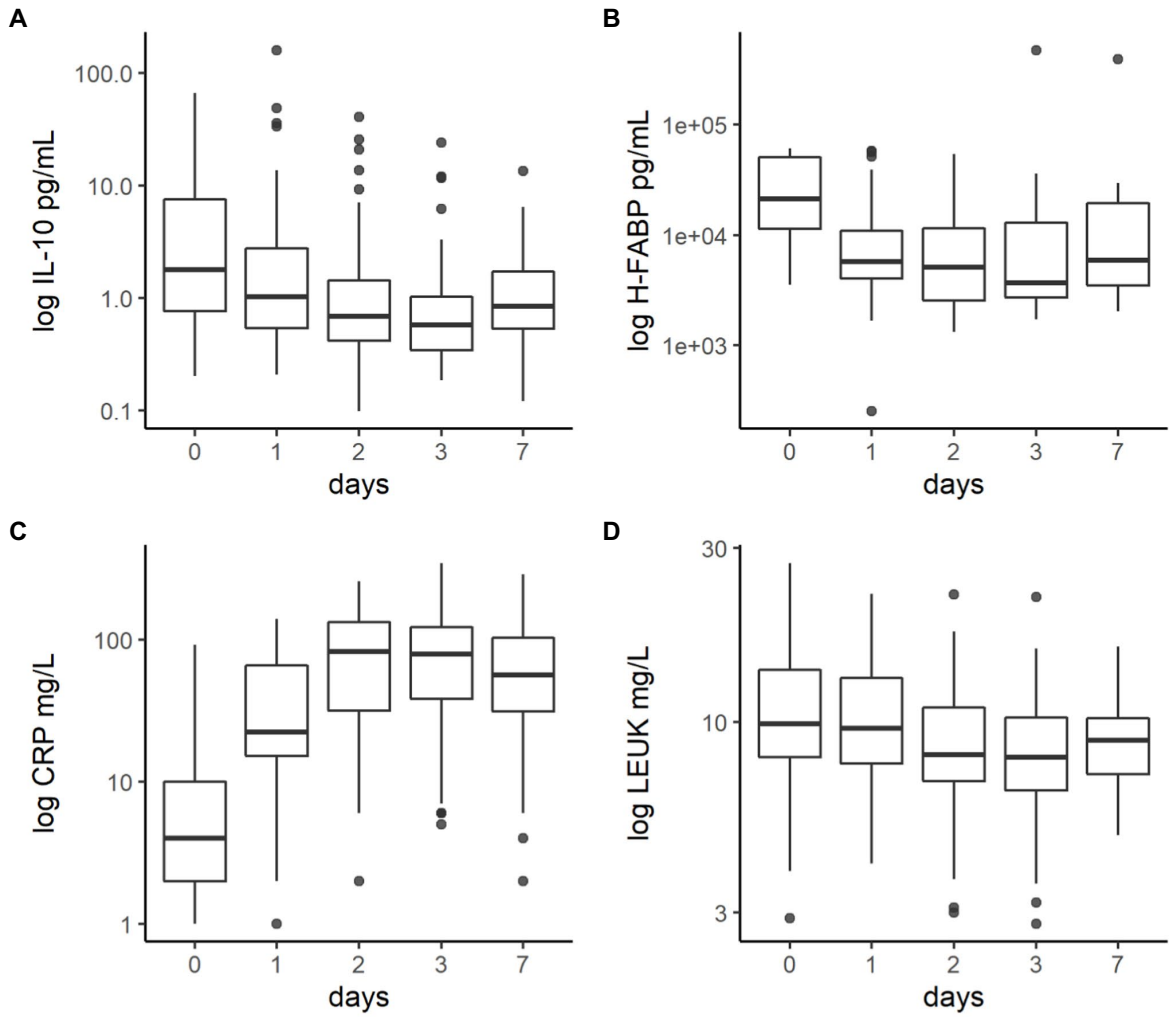
**(A)** The concentration of IL-10, **(B)** H-FABP, **(C)** CRP and **(D)** leuk during the first week after traumatic brain injury. The line that divides the box into two parts is median. The bottom of the box represents first quartile (Q1) and the top of the box represents third quartile (Q3). The lower whisker represents a minimum score (excluding outliers) and the upper whisker represents a maximum score (excluding outliers).

**Figure 2:**
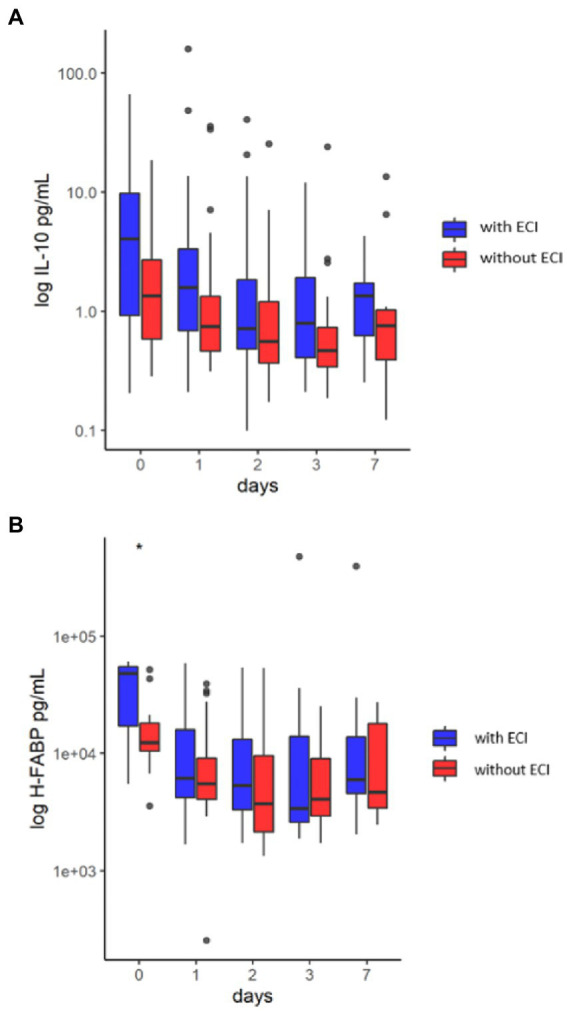
**(A)** Boxplot of IL-10 concentration in the first week after traumatic brain injury in the groups of patients with and without extracranial injuries. There was no significant difference in the concentration of IL-10 between traumatic brain injury patients with and without extracranial injuries. The line that divides the box into two parts is median. The bottom of the box represents first quartile (Q1) and the top of the box represents third quartile (Q3). The lower whisker represents a minimum score (excluding outliers) and the upper whisker represents a maximum score (excluding outliers). **(B)** Boxplot of H-FABP concentration in the first week after traumatic brain injury in the groups of patients with and without extracranial injuries. There was a significant difference in the concentration of H-FABP between traumatic brain injury patients with and without extracranial injuries on day 0 (*). The line that divides the box into two parts is median. The bottom of the box represents first quartile (Q1) and the top of the box represents third quartile (Q3). The lower whisker represents a minimum score (excluding outliers) and the upper whisker represents a maximum score (excluding outliers).

Statistical analysis was performed using R (http://www.rproject.org, versions 4.0.3 and 4.1.1) in RStudio (http://www.rstudio.com, versions 1.2.5033 and 2022.2.3.492). The levels of IL-10, H-FABP, CRP, and leukocyte counts are log-transformed in boxplots (Figures 1, 2) because of broad fluctuation of these biomarker levels. Therefore, a log scale in labels denotes the actual levels of biomarkers measured.

Paired Samples *t*-test was used to compare the demographics of the patients. Kolmogorov–Smirnov test was used for investigating the normality of distribution for the protein levels. All proteins were non-normally distributed, which is why non-parametric tests were used. Differences between the patients with and without ECI were analyzed using Mann–Whitney U-test. Correlations of IL-10 and H-FABP with CRP and leukocytes and admission correlation of H-FABP with TnT, CK, and CK-MBm were analyzed using Spearman correlation. Statistical significance was inferred at value of *p* < .05.

Correlation matrixes of IL-10, H-FABP, TnT, CK, CK-MBm, age, HCTS, and ISS were investigated with all patients, patients with ECI, and patients without ECI. Because of multiple testing, FDR (False Discovery Rate) value was used to adjust *p*-values in multiple testing. A multivariable logistic regression analysis was performed to examine whether GCS, pupillary reactions, ISS, and HCTS are associated with IL-10 and H-FABP levels upon admission.

## Results

There were 82 patients with mo/sTBI, but 12 patients lacked blood samples, leaving 70 patients with available biomarkers. There were 56 males (80%) and 14 females (20%), with a mean age of 53.7 ± 18.2 years. There were 32 patients (46%) without ECI and 38 patients (54%) with ECI. There were 30 patients with sTBI (43%) and 40 patients with moTBI (57%) in the study cohort. Demographic data are presented in Table 1.

**Table 1:** Demographics of moderate and severe TBI subgroups and subgroups of patients with/without ECI.

	Both severities (*n* = 70)	Without ECI (*n* = 32)	With ECI (*n* = 38)	*p*-value
Age (mean ± SD)	53.7 ± 18.2	56.3 ± 14.8	51.5 ± 20.5	0.25
Age (min–max)	19–91	19–76	19–91	
Sex (male/female)	56/14	25/7	31/7	
ISS (mean ± SD)	20.2 ± 13.3	15.9 ± 9.5	24.0 ± 15.1	**0.01**
sTBI/moTBI	30/40	12/20	18/20	

*p*-values < 0.05 are bolded.

### Concentrations of biomarkers, CRP, and leukocytes during the first week after injury

The concentrations of IL-10 and H-FABP, as well as CRP and leukocyte counts during the first week after TBI are shown in Tables 2–5 and Figure 1. IL-10 was highest upon admission and decreased after the first day. H-FABP showed a greater variation in trajectory after trauma, as shown in Tables 2–5. The CRP levels were near zero upon admission but increased during days 1 and 2. The leukocyte counts peaked immediately after injury and decreased constantly during days 1 and 2.

**Table 2:** Levels of IL-10 in days 0–7 within the study cohort (pg/ml).

	*N*	Median	Min	Max	IQR
IL-10, day 0	31	1.79	0.20	66.23	6.92
IL-10, day 1	62	1.03	0.21	159.06	2.24
IL-10, day 2	62	0.69	0.10	40.57	1.02
IL-10, day 3	41	0.58	0.19	24.08	0.68
IL-10, day 7	29	0.85	13.55	13.55	1.18

**Table 3:** Levels of H-FABP in days 0–7 within the study cohort (pg/ml).

	*N*	Median	Min	Max	IQR
H-FABP, day 0	31	21253.15	3547.90	60861.40	39674.92
H-FABP, day 1	62	5736.61	253.43	58359.08	6951.68
H-FABP, day 2	62	5077.96	1328.05	53982.72	9067.55
H-FABP, day 3	41	3671.96	1711.99	473948.40	10233.34
H-FABP, day 7	29	5903.80	2029.16	393977.46	16058.18

**Table 4:** Levels of CRP in days 0–7 within the study cohort (mg/L).

	*N*	Median	Min	Max	IQR
CRP, day 0	67	1	0	92	6
CRP, day 1	68	22	0	140	49
CRP, day 2	68	83	2	260	102
CRP, day 3	62	79	5	348	85
CRP, day 7	38	57	2	291	73

**Table 5:** Levels of leukocytes in days 0–7 within the study cohort (mg/L).

	*N*	Median	Min	Max	IQR
Leuk, day 0	69	9.90	2.90	27.30	5.9
Leuk, day 1	69	9.60	4.10	22.50	5.5
Leuk, day 2	68	8.15	3.00	22.40	4.1
Leuk, day 3	63	8.00	2.80	22.00	3.8
Leuk, day 7	39	8.90	4.90	16.10	3.05

### IL-10 and H-FABP in patients with and without ECI

The trajectories of IL-10 and H-FABP are displayed as boxplots in Figure 2. There was no difference in the levels of IL-10 between patients with and without ECI at any timepoint. The levels of H-FABP differed between patients with and without ECI on admission day (48.2 ± 20.5 vs. 12.4 ± 14.7 ng/ml, respectively, *p* = 0.02).

### Biomarker correlations with CRP and leukocytes

A positive correlation of IL-10 with CRP was detected on day 2 (R = 0.43, *p* < 0.001) and day 7 (R = 0.46, *p* = 0.03). There was no correlation between IL-10 and leukocyte counts at any timepoint. A negative correlation of H-FABP with CRP was detected on admission day (R = -0.45, *p* = 0.01). There was no correlation between H-FABP and leukocyte counts at any timepoint. The correlations of IL-10 and H-FABP with CRP and leukocytes are presented in Tables 6–9.

**Table 6:** Correlation of IL-10 with CRP.

	Coef correlation (Rho Spearman)	*p* value
Day 0	−0.26	0.16
Day 1	0.21	0.11
Day 2	0.43	**<0.001**
Day 3	0.16	0.32
Day 7	0.46	**0.03**

*p* values < 0.05 are bolded.

**Table 7:** Correlation of IL-10 with leukocytes.

	Coef correlation (Rho Spearman)	*p* value
Day 0	0.16	0.38
Day 1	0.06	0.66
Day 2	0.09	0.49
Day 3	0.27	0.09
Day 7	−0.08	0.73

**Table 8:** Correlation of H-FABP with CRP.

	Coef correlation (Rho Spearman)	*p* value
Day 0	−0.45	**0.01**
Day 1	−0.08	0.56
Day 2	−0.16	0.21
Day 3	0.03	0.87
Day 7	−0.11	0.61

*p* values < 0.05 are bolded.

**Table 9:** Correlation of H-FABP with leukocytes.

	Coef correlation (Rho Spearman)	*p* value
Day 0	0.22	0.22
Day 1	0.04	0.75
Day 2	0.07	0.61
Day 3	−0.22	0.17
Day 7	0.05	0.82

### Correlations between H-FABP and the levels of TnT, CK, and CK-MBm on admission

**Figure 3:**
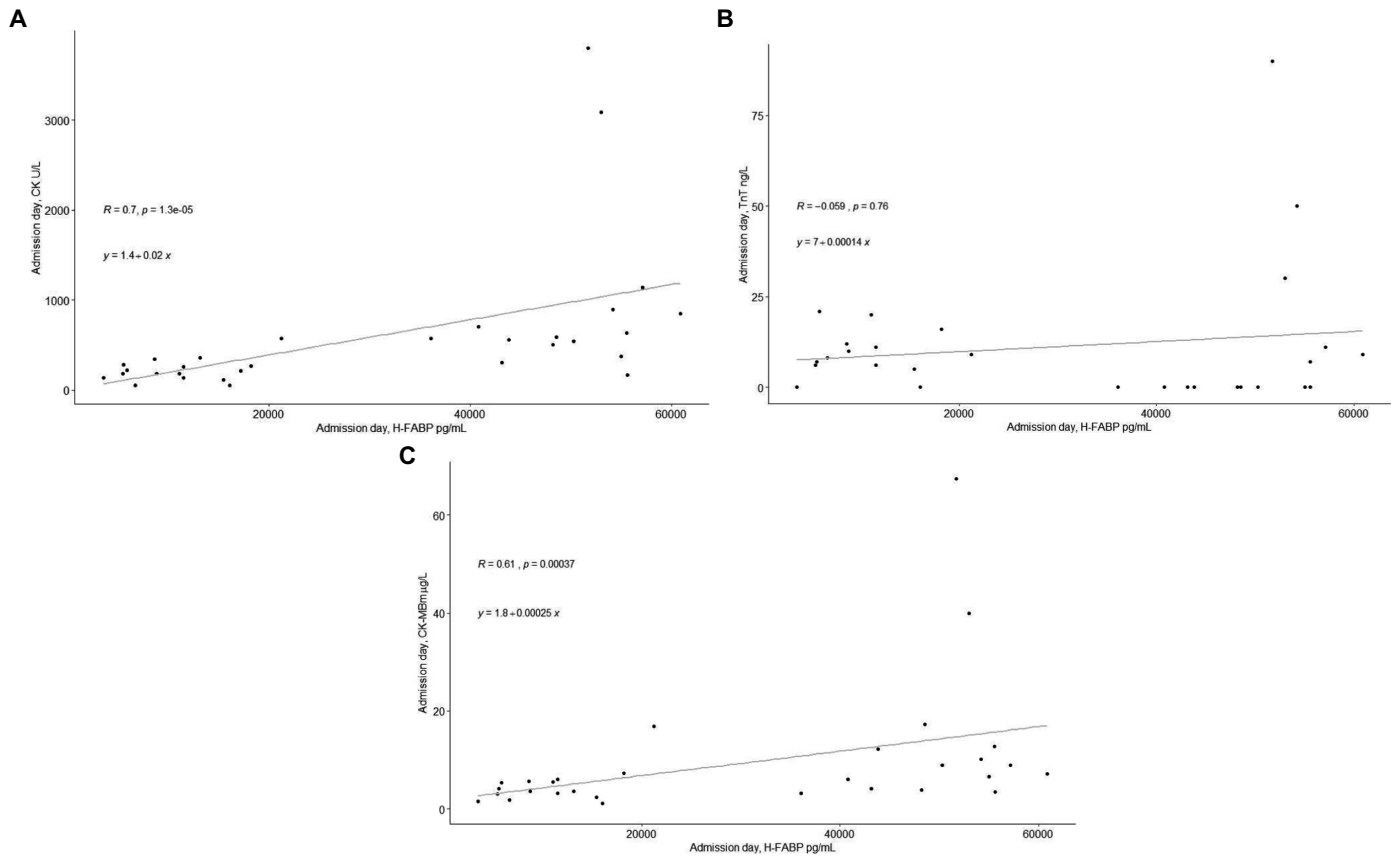
**(A)** The correlation between admission level of H-FABP with admission level of CK. **(B)** The correlation between admission level of H-FABP with admission level of TnT. **(C)** The correlation between admission level of H-FABP with admission level of CK-MBm.

There was no correlation between H-FABP and TnT upon admission. There were significant positive correlations between H-FABP and CK and CK-MBm (R = 0.70, *p* < 0.001 and R = 0.61, *p* < 0.001, respectively). The correlations of H-FABP with admission TnT, CK and CK-MBm are presented in Figure 3.

### The associations of age, GCS upon admission, pupil reactions, ISS, and HCTS with the IL-10 and H-FABP levels

A multivariable regression analysis was performed to analyze the associations of age, admission GCS, pupillary reactions, ISS, and HCTS with the IL-10 and H-FABP levels. No significant associations were found with the levels of IL-10. A significant association was found between the levels of H-FABP and ISS (*p* = 0.03).

## Discussion

This study of patients with mo/sTBI investigated the trajectories of IL-10 and H-FABP in patients with and without ECI. We also studied whether there are differences in the levels of IL-10 and H-FABP between patients with and without ECI. Furthermore, we studied whether inflammatory reactions and/or extracranial injuries affect the levels of IL-10 and H-FABP in patients with mo/sTBI. There was a difference in the levels of H-FABP between patients with and without ECI on day 0. In contrast, there was no difference in the levels of IL-10 between patients with and without ECI. There was no correlation between leukocyte count and IL-10 and H-FABP levels. Correlations were found between CRP and IL-10 and between CRP and H-FABP levels. There was a correlation between H-FABP and CK/CK-MBm levels at admission, but not between H-FABP and TnT levels at admission.

Earlier studies have investigated the trajectories of different TBI biomarkers, but studies on IL-10 and H-FABP are scarce. IL-10 has been shown to peak rapidly after trauma and decrease during the next few days after trauma (7). Our results are in line with these findings. The current results suggest that H-FABP levels do not peak as pronouncedly and that H-FABP levels fluctuate more compared to IL-10, possibly due to extracerebral sources.

There was a difference in the levels of H-FABP between patients with and without ECI on the day of admission, suggesting that extracranial injuries may affect the levels of H-FABP. The observed association between H-FABP and ISS supports this suggestion. Also, an earlier study has suggested that the levels of H-FABP are higher in patients with multi-trauma (20). H-FABP is also a protein expressed in cardiac myocytes (8) and it is possible that H-FABP leaks from cardiac tissue in patients with multi-trauma. There were no differences in the levels of IL-10 between patients with and without ECI. This finding is in line with other studies (21, 22).

In order to improve understanding the usability of IL-10 and H-FABP in TBI diagnostics, it is important to know how their levels in circulation compare with inflammatory and cardiac biomarkers. High-sensitivity C-reactive protein (hs-CRP) may be a promising prognostic protein for predicting disability after a TBI, and the levels of hs-CRP have been higher in patients with poor outcome (23). On the other hand, the levels of CRP do not appear to correlate with acute GCS score after TBI when measured using standard CRP (24). In our study, we found that levels of standard CRP were correlated with both IL-10 and H-FABP levels at a few timepoints. These results are not comparable with studies using hs-CRP because of the different concentration ranges and because hs-CRP detects lower protein levels than standard CRP. Therefore, further studies on correlations between hs-CRP and IL-10 or H-FABP are needed. We found no correlation between the leukocyte counts and IL-10 or H-FABP levels. Previous studies of leukocyte levels in TBI are few, but they may be a predictive biomarker after TBI in pediatric patients (25).

Cardiac dysfunction during the early days after a TBI (26) has been observed using cardiac echocardiogram and high sensitivity troponin levels (27). A correlation analysis between admission H-FABP and cardiac enzymes was performed to evaluate the possible cardiac involvement in H-FABP levels. The admission levels of H-FABP correlated with CK and CK-MBm but not with the levels of TnT. We were not able to rule out a cardiac source of H-FABP as our patients with TBI did not have a cardiac echocardiogram or high sensitivity troponin assessment available. Combining these in future studies in patients without ECI could show if H-FABP has also cardiac origin after a TBI.

This study has limitations. The study population was relatively small, which increases the risk of overfitting bias, why further studies with larger study cohorts are needed. Secondly, the study population contained only 14 female patients, therefore reliable statistical analyses between the genders were impossible to do. Furthermore, the time interval between the admission samples and TBI (<24 h) might be too long for several biomarkers with a short half-life such as H-FABP and IL-10. Moreover, we also lacked some blood samples from our study patients from the first week after TBI.

## Conclusion

Our results suggest that inflammatory reactions during the early hospital stay after a TBI do not significantly confound the use of IL-10 or H-FABP as possible TBI biomarkers. Extracranial injuries, by contrast, may significantly affect the levels of H-FABP. The correlations between H-FABP and CK/CK-MBm and the association between H-FABP and ISS suggest that cardiac sources and extracranial injuries may influence the levels of H-FABP in patients with TBI. Further studies with larger study cohorts on these topics are needed.

## Data availability statement

The original contributions presented in the study are included in the article/supplementary material, further inquiries can be directed to the corresponding authors.

## Ethics statement

The studies involving human participants were reviewed and approved by Southwest Finland Hospital District Research Ethics Committee (decision 68/180/2011). The patients/participants provided their written informed consent to participate in this study.

## Author contributions

TN: manuscript writing, methodology, and statistical analysis. A-CC: statistical analysis and manuscript revision. RT: recruiting patients, data curation, manuscript drafting, and manuscript revision. MV: statistical analysis. OT: organizing and planning of the study and manuscript revision. VN: manuscript revision and original TBI care study design. H-RM: recruiting patients and manuscript revision. JT: recruiting patients and manuscript revision. MM: manuscript revision. IH: data curation and manuscript revision. MG, DM, and PH: original TBI care study design and manuscript revision. J-CS: biomarker analysis and manuscript revision. JP: recruiting patients, data curation, planning of the study, manuscript drafting, and manuscript revision. All authors contributed to the article and approved the submitted version.

## Funding

This work was partially funded by the Neurocenter, Turku University Hospital (TN), the University of Turku (TN), Academy of Medical Sciences/The Health Foundation Clinician Scientist Fellowship and National Institute of Health and Care Research (NIHR) Advanced Fellowship (VN), The Finnish Medical Foundation (IH), The Päivikki and Sakari Sohlberg Foundation (IH), The Paulo Foundation (IH), the Finnish Cultural Foundation (IH), The Academy of Finland (JP, Grant 17379), and the Maire Taponen Foundation (JP).

## Conflict of interest

The authors declare that the research was conducted in the absence of any commercial or financial relationships that could be construed as a potential conflict of interest.

## Publisher’s note

All claims expressed in this article are solely those of the authors and do not necessarily represent those of their affiliated organizations, or those of the publisher, the editors and the reviewers. Any product that may be evaluated in this article, or claim that may be made by its manufacturer, is not guaranteed or endorsed by the publisher.
